# Problematic Relationships with Smartphones of Spanish and Colombian University Students

**DOI:** 10.3390/ijerph17155370

**Published:** 2020-07-25

**Authors:** Verónica Marín-Díaz, Juan Manuel Muñoz-González, Begoña-Esther Sampedro-Requena

**Affiliations:** Education department, University of Cordoba, 14071 Córdoba, Spain; juan.manuel@uco.es (J.M.M.-G.); bsampedro@uco.es (B.-E.S.-R.)

**Keywords:** problematic smartphone use, university student, internet, MPPUSA

## Abstract

The presence of smartphones in the lives of the population in general, and of youth in particular, is evident, and is derived from elements such as the diversity of prices as well as the ease of access of all the resources that can be reached through the internet. With the use of a descriptive approach using a quantitative poll, the objective of the present study was to discover the opinions of university students in Spain and Colombia about smartphone use, as well as the consequences of its use, and if this use could derive into so-called problematic smartphone use (PSU). For gathering the information, the *Mobile Phone Problematic Use Scale* (MPPUSA) was utilized, with a sample size *n* = 4009. The main result reached was that the model obtained is structured around six factors that determined the elements in light of PSU. The initial conclusion found was that the model applied can be utilized with Colombian students, with young Spanish women and students in the macro area of Social Sciences, the ones who had problematic behavior with the devices, as compared to the Health Sciences students who did not have it.

## 1. Introduction

It is a known fact that the present society is digital and that the so-called Information and Communication Technologies (from here on ICT) coexist with us systematically, allowing for the interactions with fellow humans to occur at anytime and anywhere [1]. All of this is thanks to the universalization of the internet in general, and the tools which we can use through it, as these tools provide abundant means for socializing with others, perform consultations of any kind, search for knowledge, entertain ourselves, etc. [1,2,3].

The IWS [4] points out that in 2019, there were more than 4 billion people connected in the world, and in the specific case of Spain [5] this figure was the non-negligible amount of 42.4 million users and in Colombia more than 31 million people from a population of more than 49 million [4,6]. In both countries, 96% and 63% of users accessed the internet through their smartphones, respectively.

Nowadays, independently of the country where we live, the smartphone is one of the key technologies for the population at large. The diversity of the possible functions of these devices, apart from the use for which they were originally conceived [7], has made them become a key feature in the processes of socialization and learning of children, adolescents and young adults. As indicated by many studies [8,9,10,11,12], these devices allow accessing a great variety of content presented in different formats, messaging services and social networks, allowing for the development of different skills and competencies beyond the digital, allowing access to other technological resources, etc., which makes them even more attractive.

Related to this, diverse studies [13,14] have shown that Spanish university students, most of whom are younger than 35 years old, use smartphones from a social, academic and personal point of view. Likewise, for Colombian university students [15], smartphone use has different aims, such as navigating on the internet, or checking e-mail. It is unquestionable that the use of this device has increased around the world in the last few years, especially in the population within this age range (university students younger than 35). This has extended its use to the area of education, where some studies [16] have shown the student’s habits within and outside university spaces, where in the first scenario it is commonly used in transportation and outdoors; while in the second scenario the classrooms, the hallways and the libraries are the most exploited places. It is precisely the attractiveness they possess that has resulted on their use increasing exponentially, with some authors [3,17,18,19] already speaking about the problematic use of smartphones.

Before continuing, it should be mentioned that within the scientific community, there is no clear definition of this behavioral disorder, as we have found authors who discuss the problematic use of the device [3,17,18,19], while others prefer to talk about addiction [1,2,20,21,22]. However, the purpose of this study is not to delve into this debate, as the starting position of this work is that only a problematic relationship exists [17,18], detailed on the fifth version of the *Diagnostic and Statistical Manual of Mental Disorders* [23] which points to this relationship not having an addiction factor, given that this publication only mentions addiction in cases of pathological gambling and a behavioral disorders. Thus, it is understood that only a problematic use of the device exists, indicating that a medical condition recognized by the entire community does not exist [11].

On the other hand, some authors [17,24,25,26,27] point to factors such as the lack of sleep, hyperactivity, insecurity when driving, anxiety, depression, personality disorders, etc., associated to harmful behaviors such as in the use drugs including heroin, alcohol, cannabis, speed, etc., that although not linked to technologies, have been observed in the population who have a problematic use of mobile phones. Nevertheless, some research studies [28] suggest that there is a common factor among the addictive behaviors in the use of substances and the internet, the impulsivity which is manifested, among other aspects, by low academic performance or low levels of concentration, however, this cannot be extrapolated to an addictive use of the smartphone.

At the same time, it should be pointed out that as [11] indicate, the population, who along with adolescents, are the main population at risk of having a problematic use of the devices, are the university students, given that they consider the device as part of them [11,24]. On the other hand, the fear of being left out of relationships and developing the so-called FOMO syndrome [29], makes this population group more susceptible to developing a behavioral disorder, which is translated into the problematic use of the phone.

Mobile phones, as previously indicated [1,2,3,30], provide their users with numerous gratifications which begs the question of what type of relationship we as individuals have developed towards them or with them. Some research studies have determined that a variable that determines the presence of problematic use is the amount of time spent utilizing the devices [11,30,31], while others indicate that the variable gender is the one that defines this relationship, with women showing a greater relationship with them. Thus, works such as [32] point out that younger people aged 18–29 years old have a high dependency on their mobile phones, which can lead to the individuals developing an emotional support relationship [18]. These aspects, in the education sphere for example, have resulted in some education centers prohibiting their presence in the centers [8].

As a result and given the high number of connections produced with smartphones in Spain and in Colombia [4,5,6], it is necessary to clarify the attitude of university students towards these devices. In this sense, the main objective of the present research, which will be described below, is to discover the opinions of the university students in Spain and Colombia on the use of smartphones, as well as the consequences of this use. Studies conducted among Spanish and Colombian university students [8,12,17,18] have brought to light that this scale fits the smartphone user profile, as well as the needs of the context.

The main conclusions found are: on the one hand, that younger students show a problematic use of smartphones [18,32], and in general, women more frequently display this behavioral disorder [11,30,31], without differences also found as a function of the country of origin.

The main objective of the research was to analyze the behavior of the six MPPUSA factors according to sex, age, country, macro area and self-perception in the problematic use of the smartphones by university students from Spain and Colombia. This general objective, at the same time, is concretized in the following specific objectives:

- To discover the existence of a problematic use of the mobile phone in Spanish and Colombian university students.
- To analyze if there are differences between the students objects of the study with respect to the problematic use of the mobile phone as a function of their age and sex, as well as their country of origin and macro area.
- To verify if there are relationships between the different dimensions of the questionnaire
- To explain which factors have an influence on the perceptions of the students of the problematic use of mobile phones, as well as its consideration as a function of the country, of their age and sex.

## 2. Materials and Methods

The design of the study utilized a descriptive approach with a cross-sectional, quantitative survey, due to the numerical and reliable nature of the data collected, and the use of a deductive and structured research strategy.

### 2.1. Sample

To select the sample, non-probabilistic, convenience sampling [33] was utilized, given that the questionnaire was provided to the students whom the study researchers had access to during 2017–2018 academic year.

The sample was comprised by a total of 4009 students, of which 60.1% were female and 39.9% male, distributed in the following manner: 2965 were enrolled in the National Open and Distance University (UNAD), Colombia (74.0%) and 1044 from the University of Cordoba, Spain (26%). As for the student profiles, the macro area of enrollment was considered, addressing the five university macro areas established in both countries, which were: Social and Judicial Sciences (S and JS, *n* = 1911, 47.7%), Health Sciences (HS, *n* = 726, 18.1%), Arts and Humanities (A and H, *n* = 164, 4.1%), Experimental Sciences (ES, *n* = 168, 4.2%), and Engineering and Architecture (E and A, *n* = 1039, 25.9%), as well as age, as shown in Table 1.

### 2.2. Instrument

The data collection instrument utilized in this research was based on the Mobile Phone Problematic Use Scale for Adolescent (MPPUSA) [34], it is contextualized to the Spanish-speaking population. This questionnaire is anonymous, with closed-ended questions and polythematic, with a Likert-type response scale, ranging from complete disagreement (1) to complete agreement (5). The factorial structure was determined through an Exploratory Factorial Analysis (EFA), using the principal components method for extracting the factors, with the following indices of adjustment: KMO = 0.98, Bartlett’s sphericity test with *p* < 0.001, and a total explained variance of 56.83%. The classification of the MPPUSA items by [35] was used as a reference providing the following structure with six dimensions as a result:

- Tolerance (Cronbach’s alpha = 0.904): this dimension alludes to aspects related with the usage time of the smartphone and the impossibility of decreasing it. It encompasses a total of three items.
- Escape route (Cronbach’s alpha = 0.907): comprised by three items, it alludes to the device as a resource for evading specific problems. It encompasses elements such as the increase in the feeling of well-being, its use when feeling lonely, as well as its prioritization against urgent matters or tasks.
- Disconnection (Cronbach’s alpha = 0.912): this dimension covers aspects such as the appearance of worry if the subject cannot answer a call, the inability of disconnecting the mobile phone or the appearance of the feeling of disorientation in the case of not having the device available.
- Anxiety (Cronbach’s alpha = 0.919): composed by three items that allude to the inability of the subjects to reduce the smartphone usage time, the appearance of nervousness in the case of not being able to read messages or answer calls, as well as the anger when they have the obligation of turning the device off.
- Negative consequences (Cronbach’s alpha = 0.935): it constitutes the broadest dimension of the questionnaire, as it encompasses a total of 12 items that refer to the prioritization of the use of the smartphone against other tasks, the reduction of hours of sleep of the subjects, the making of economic investments beyond their means, the appearance of tardiness, the appearance of inconveniences from its use or the decrease in academic and/or professional performance, among other aspects.
- Social motivations (Cronbach’s alpha = 0.901): this dimension alludes to aspects such as the impossibility of maintaining communication with one’s peers in the case of not having the device, as well as the perception of anger by them in the case that the terminal is not connected; it encompasses two items.

As for reliability, it was measured through the internal consistency approach, obtaining a Cronbach’s alpha value of > 0.90, for the dimensions as a set, as well as individually, demonstrating the high reliability of the instrument.

Lastly, an item with a dichotomous answer (yes/no) was included about the self-perception they considered they had about the problematic use of Smartphones (explaining it according to [19], as a persistent deterioration or anguish), formulated in the following terms: «I believe I have a problematic use of the Smartphone», as well as a series of independent variables from the academic area (macro area) and sociodemographic areas (age, gender and country).

Lastly, the instrument had a total of 26 items written as statements and structured into the six dimensions previously mentioned, 

### 2.3. Procedure

The filling of the questionnaire was done in person and anonymously, with the study researchers present in the classroom to help answer any possible doubts of the students in the understanding of the items in the instrument. In the case of the Colombian students, a videoconference system was established, which allowed for the resolution of problems in real time during the completion of the instrument.

### 2.4. Analysis Conducted

In this study, the following analysis were utilized to reach the objectives set:

First, a descriptive analysis of the 26 variables of the questionnaire was performed, through the measurements of central tendency (mean) and dispersion (standard deviation). And the variable of the self-perception of the problematic use of the Smartphone.

In second place, a descriptive analysis was performed of the six dimensions of the questionnaire, which was similar to the analysis described above. In third place, an analysis of variance was conducted to verify the existence or not of differences in each of the dimensions of the questionnaire as a function of the independent variables (country, gender, age and macro area), through Student’s *t*-test and ANOVA (both with n.s = 0.05). Afterwards, the relationship between the dimensions that comprised the questionnaire were verified through bivariate correlations.

Lastly, binary logistic regressions were utilized with a forward selection Wald’s test and considering the goodness-of-fit of Hosmer-Lemeshow and the Confidence Interval (CI) for exp(B), to explain the influence of the six factors studied on the self-perception of the problematic use of the smartphone and the influence of the country.

All of these analyses were performed with the SPSS statistical package, version 23 (IBM Corp., Armonk, NY).

## 3. Results

### 3.1. Descriptive Study

In first place, the descriptive results (mean and standard deviation) of the 26 items that compose the *MPPUSA Questionnaire* used in this research study are listed (see Table 2).

It can be observed that the items 5, 9, 10 and 16 point to being more in agreement with the statements as compared with items 4, 14, 21 and 22, which point to being more in disagreement.

Likewise, the results obtained in the self-perception on the problematic use of the Smartphone, point that 79.9% (*n* = 3205) considered that they did not have a problematic use, as compared to 20.1% (*n* = 804) who did.

#### 3.1.1. Tolerance

The results obtained highlight that the students were indifferent as for the tolerance in the use of the smartphone (M = 3.00, SD = 0.87).

With respect to the comparison of means related with country and gender, the Student’s *t*-test for independent samples showed statistically significant differences in the first case, as *t* = 6.167, *p* < 0.050, with the Colombian students being the ones who obtained a greater score in this dimension (M = 3.04 vs. M = 2.87).

Lastly, for age and the macro area, the analysis of variance (ANOVA) only pointed to the existence of statistically significant differences in the second case, as [F (4, 4003) = 5.373; *p* < 0.050]. The post hoc multiple comparisons using the Games-Howell test showed within which macro area the differences in means were found. These macro areas were Experimental Sciences along with Engineering and Architecture, with respect to Social and Judicial Sciences, which had the greatest score (μ = 3.13 and y μ = 3.05 vs. μ = 2.93).

#### 3.1.2. Escape Route

Once the analysis was performed, the results pointed out that the students were partially in disagreement as for the use of the smartphone as an escape route (M = 2.90, SD = 0.94).

As for the comparison of means, the Student’s *t*-test for independent samples provided evidence of the existence of statistically significant differences for the country, exclusively (*t* = 2.262, *p* < 0.050), with the mean for the Colombian subjects being higher than the Spanish ones (2.91 vs. 2.85).

Lastly, for age and macro area, the analysis of variance (ANOVA), showed statistically significant differences in the first case [F (3, 4005) = 20.038; *p* < 0.050], with the difference of means found in students older than 26 with respect to those aged 18 to 20, 21 to 23, 24 to 26, respectively (μ = 2.74 vs. μ = 3.01, μ = 2.97 and μ = 2.89).

#### 3.1.3. Disconnection

The results obtained showed that the students were partially in disagreement with respect to the disconnection of the mobile phone (M = 2.63, SD = 1.09).

The Student’s *t*-test for independent samples, performed to detect possible differences with respect to the country and gender, highlighted statistically significant differences in both cases. In the first (*t* = −3.577, *p* < 0.050), the Spanish students obtained a greater score with respect to the Colombian students (M = 2.73 vs. M = 2.60). As for gender (*t* = −3.456, *p* = 0.001), women were the ones who obtained a greater score with respect to the men (M = 2.68 vs. M = 2.56).

On the other hand, the analysis of variance (ANOVA) utilized to find statistically significant differences between age and the macro area, showed statistically significant differences in both cases, with the students older than 26 obtaining the lowest score [F (3, 4005) = 6.778; *p* = 0.000], as compared to those aged 18–20, 21 to 23 and 24 to 26, respectively (μ = 2.54 vs. μ = 2.71, μ = 2.70 and μ = 2.58). On the other hand, as for the macro area [F (4, 4003) = 2.634; *p* < 0.050], differences were found in the area of Social Sciences with respect to Health Sciences, with this last obtaining the lowest score (μ = 2.54 vs. μ = 2.68).

#### 3.1.4. Anxiety

The results obtained highlight that the students had an opinion that was partially in disagreement for the anxiety dimension of the use of the smartphone (M = 2.19, SD = 1.04).

With respect to the comparison of means related with country and gender, Student’s *t*-test for independent samples showed statistically significant differences only in the second case, as *t* = −2.225, *p* = 0.020, with the women receiving a higher score in this dimension (M = 2.22 vs. M = 2.14).

Lastly, for age and the macro area, the analysis of variance (ANOVA) only pointed to the existence of statistically significant differences in the first case (age), as [F (3, 4005) = 7.180; *p* = 0.000]. The multiple post-hoc comparisons with the Games-Howell test allowed us to find exactly in what age range were these differences in means found, with those older than 26 being the ones who had a lower score with respect to students with ages between 18 and 20, and 21 and 23, respectively μ = 2.09 vs. μ = 2.28 and μ = 2.21)

#### 3.1.5. Negative Consequences

Once the analysis was performed, the results showed that the students were partially in disagreement about the negative consequences derived from the use of the smartphone (M = 2.31, SD = 0.84).

For the comparison of means, Student’s *t*-test for independent samples showed the existence of statistically significant differences for country, exclusively (*t* = 8.891, *p* = 0.000), with the means for the Colombian students being higher than for the Spanish ones (2.36 vs. 2.14).

Lastly, for the age and the macro area, the analysis of variance (ANOVA) indicated statistically significant differences in both cases. In the first case [F (3, 4005) = 13.181; *p* = 0.000], finding the differences in means of the students aged between 18 and 20, with respect to the rest (μ = 2.42 vs. μ = 2.30, μ = 2.29 and μ = 2.21), and in the second [F (4, 4003) = 9.024; *p* = 0.000], the differences in means were found in the students belonging to the macro area of Social and Judicial Sciences with respect to those in Arts and Humanities and Engineering and Architecture, respectively (μ = 2.23 vs. μ = 2.48, μ = 2.39).

#### 3.1.6. Social Motivations

The results obtained showed that the students were partially in disagreement with respect to the social motivations derived from the use of the mobile phone (M = 3.22, SD = 1.03).

Student’s *t*-test for independent samples, performed to detect possible differences with respect to country and gender, highlighted statistically significant differences only in the first case, *t* = 5.079, *p* = 0.000, with the Colombian students the ones who had the highest scores as compared to the Spanish ones (M = 3.27 vs. M = 3.09).

Also, the ANOVA performed to verify statistically significant differences between age and macro area, showed that there were no statistically significant differences in any of the cases.

### 3.2. Correlational Analysis

This section addresses the correlational study between the six dimensions of the questionnaire. The data obtained, after the use of a Spearman’s correlation test to observe the relationship between the six dimensions of the scale, can be observed below (see Table 3):

As a function of the data obtained, it is observed that all the dimensions were correlated amongst themselves, with these correlations being high and moderate [36], using as a reference the value of their respective coefficients. In this sense, the following had a high correlation: the dimension Disconnection with the dimension Anxiety, as Rho = 0.754 and *p* = 0.000; as well as this last with the dimension Negative consequences (Rho = 0.678 and *p* = 0.000). As for moderate correlations (as defined by the authors cited previously), these were observed in the rest of the dimensions, as they obtained values between 0.300 and 0.600.

### 3.3. Binary Logistic Regression Analysis

The best fitting model with respect to the self-perception shown by the university students from Spain and Colombia on the problematic use they display, was produced through a binary logistic regression. As this independent dependent variable is dichotomous in nature, with Yes/No values, the forward selection Wald test was utilized [37]. This model had a high specificity (98.8%) and high sensitivity (95%), with an overall percentage of 98% for the cut-off value of 50%, which indicates that the model classifies equally well those who consider themselves to have a self-perception on the problematic use of the Smartphone as well as those who do not.

The results shown from the analysis point out, for a sample of 4009 subjects, where 3205 answered No (79.9%) and 804 Yes (20.1%), that the goodness-of-fit of the best fitting model shows: a -2log of likelihood = 354.509, a Cox and Snell R^2^ = 0.599; and an R^2^ of Nagelkerke = 94.6% (0.946); aside from the value of the Hosmer and Lemeshow test which shows a good fit, as its significance is 0.988 (≥0.05 according to [38]). Likewise, the values obtained in the Chi-square test for step (χ^2^ = 3663.81, gl = 6 and *p* < 0.05); block (χ^2^ = 3663.81, gl = 6 and *p* < 0.05); and model (χ^2^ = 3663.81, gl = 6 and *p* < 0.05), shows that the fitted model is significantly differentiated from the initial or base model.

As shown in Table 4, it can be observed how all the factors that appear in the MPPUSA intervene in the explanation in a significant manner. From them and tending to their relevance according to the value of B, these would be ordered as Consequences; Tolerance; Disconnection; Escape; Motivations and Anxiety.

Thus, the logistic equation would be:

Y (self-perception in the problematic use) = −89.07 + 1.13Tolerance + 1.11Escape + 1.12Disconnection + 0.54Anxiety + 1.18Consequences + 0.99Motivations(1)

Thus, it can be observed that all the factors resulting from the MPPUSA positively and significantly influence the self-perception of considering that a problem with the use of the smartphone exists (reference category).

When analyzing the explanatory model of the self-perception of the problematic Smartphone use, having in mind the country of origin as the selection variable, it is observed, for 2965 Colombian students, of which 2266 answered No (76.4%) and 699 Yes (23.6%), that the goodness-of-fit had the following values: a -2log of likelihood = 290.826; a Cox and Snell R^2^ = 0.630; and a, R^2^ from Nagelkerke = 94.8% (0.948); with the Hosmer and Lemeshow value = 0.999, which demonstrates a good fit, as its significance is ≥0.05 [38]. Also, it has a high specificity (98.5) and a high sensitivity (95%), with an overall percentage of 97.7% for the cut-off value of 50%, which indicates that the model classifies those who consider themselves to have a self-perception on the problematic use of the Smartphone as well as those who do not equally well.

Table 5 shows how all the factors obtained from the MPPUSA significantly intervene in the explanation, as it occurred in the general model, in the same order according to their relevance (B value).

In this occasion, the logistical equation is the following:

Y (self-perception on the problematic use of Colombian students) = −88.13 + 1.12Tolerance + 1.06Escape + 1.09Disconnection + 0.50Anxiety + 1.19Consequences + 0.95Motivations(2)

As a result, the self-perception of the Colombian students when considering if they have a problematic smartphone use (reference category), all the factors from the MPPUSA have a positive and significant influence.

Lastly, Table 6 shows the results of the explanatory model of the self-perception on the problematic use of the smartphone of Spanish university students (selection variable). Of the 1044 students, 939 who answered No (89.9%) and 105 Yes (10.1%). The goodness-of-fit of the best fitting model obtained the following values: a -2log of likelihood= 54.446; a Cox and Snell R^2^ = 0.451; and a Nagelkerke R^2^ = 94.2% (0.942); with the Hosmer and Lemeshow test obtaining a value of 1.0, which demonstrates a good fit, as its significance is ≥0.05 [38]. The specificity is high (99.4%), as well as its sensitivity (94.3%), with an overall percentage of 98.9%) for the cut-off value of 50%), which indicates that the model classifies those who consider themselves to have a self-perception on the problematic use of the Smartphone as well as those who do not equally well.

Thus, the equation is:

Y (self-perception of the problematic use of the smartphone of Spanish university students) = −108.94 + 1.46Tolerance + 1.48Escape + 1.08Disconnection + 1.25Anxiety + 1.27Consequences + 1.46Motivations.(3)

On Table 6, it is observed that the factors that appear in the MPPUSA significantly intervene in the explanation; however, when taking into account their relevance (B value), it is observed that the order is different from the general model and the order found for the Colombian students, being: Escape; Motivations; Tolerance; Consequences; Anxiety; Disconnection.

In summary, for the self-perception of the Spanish students who consider if they have a problem with the use of smartphones (reference category), all the factors found from the MPPUSA have a positive and significant influence.

When comparing the Spanish students with the Colombian ones, it is observed that although all the factors contribute to the best fitting models, in Spain the factor Escape had more weight than in Colombia, with Consequences being more significant for the latter.

The analysis of the explanatory model of the self-perception on the problematic Smartphone use, with sex as the selection variable, shows that for 1601 male students, of which 1270 answered No (79.3%) and 331 Yes (20.7%), the goodness-of-fit had the following values: a -2log of likelihood = 134.565; a Cox and Snell R^2^ = 0.607; and a R^2^ from Nagelkerke = 95% (0.950); with a Hosmer and Lemeshow value = 0.994. This demonstrates a good fit, as its significance is ≥0.05 [38]. Also, it had a high specificity (98.8%) and a high sensitivity (94.3%), with an overall percentage of 97.9% for the cut-off value of 50%, indicating that the model classifies both of those who consider themselves to have a self-perception on the problematic use of the smartphone as well as those who do not, equally well.

Table 7 shows that all the factors obtained from the MPPUSA significantly intervened in the explanation, as in the general model, in the same order according to their relevance (B value).

On this occasion, the logistical equation is the following:

Y (self-perception on the problematic use of male students) = −93.51 + 1.26Tolerance + 1.22Escape + 1.16Disconnection + 0.60Anxiety + 1.22Consequences + 0.90Motivations(4)

As a result, all the factors from the MPPUSA have a positive and significant influence on the self-perception of the male students when considering if they have a problematic use of the smartphone (reference category).

Table 8 shows the results of the explanatory model for female university students (selection variable). Of these 2408 students, 1935 answered No (80.4%) and 473 Yes (19.6%). The goodness-of-fit of the best fitting model had the following values: a -2log of likelihood = 215.28; a Cox and Snell R^2^ = 0.594; and a Nagelkerke R^2^ = 94.5% (0.945); with the Hosmer and Lemeshow test obtaining a value of 0.897, which demonstrates a good fit, as its significance was ≥0.05 [38]. The specificity and the sensitivity were high (98.7% and 94.3%, respectively), with an overall percentage of 97.8% for the cut-off value of 50%, indicating that the model classifies both of those who consider themselves to have a self-perception on the problematic use of the Smartphone as well as those who do not, equally well.

Thus, the equation is

Y (self-perception of the problematic use of the smartphone of female university students) = −88.4 + 1.09Tolerance + 1.03Escape + 1.11Disconnection + 0.48Anxiety + 1.19Consequences + 1.08Motivations.(5)

In Table 8, it is observed that the factors from the MPPUSA significantly intervened in the explanation; however, when taking into account their relevance (B value), it was observed that the order was different from both the general model and the one for the male students. Thus, for females, the order was: Consequences; Disconnection; Tolerance; Motivations; Escape; Anxiety.

Having in mind age as the selection variable, the explanatory model for 1237 university students aged from 18 to 20 years old, of whom 979 answered No (79.1%) and 258 Yes (20.9%), had the following goodness-of-fit values: a -2log of likelihood = 146.052; a Cox and Snell R^2^ = 0.596; and a R^2^ from Nagelkerke = 93% (0.930); with the Hosmer and Lemeshow value = 0.993, which demonstrates a good fit, as its significance was ≥0.05 [38]. Also, it had a high specificity (98.1%) and a high sensitivity (93.8%), with an overall percentage of 97.2% for the cut-off value of 50%, which indicates that the model classifies those who considered themselves to have a self-perception on the problematic use of the Smartphone as well as those who do not, equally well.

Table 9 shows how all the factors obtained from the MPPUSA significantly intervene in the explanation, as it occurred in the general model, in the same order according to their relevance (B value).

On this occasion, the logistical equation is the following:

Y (self-perception on the problematic use of smartphone of students between 18 and 20 years) = −76.99 + 0.88Tolerance + 0.95Escape + 0.99Disconnection + 0.49Anxiety + 1.03Consequences + 0.83Motivations(6)

As a result, all the factors of the MPPUSA have a positive and significant influence on the self-perception of university students aged between 18 and 20 years old when considering whether they have a problematic use of smartphones (reference category).

Table 10 shows the results of the explanatory model of students aged from 21 to 23 years old (age selection variable). Of these 860 students, 696 answered No (80.9%) and 164 Yes (19.1%). The goodness-of-fit of the best fitting model obtained the following values: a -2log of likelihood= 45.820; a Cox and Snell R^2^ = 0.602; and a Nagelkerke R^2^ = 96.7% (0.967); with the Hosmer and Lemeshow test obtaining a value of 0.995, which demonstrates a good fit, as its significance is ≥0.05 [38]. Both the specificity and sensitivity were high (99.1% and 97%, respectively), with an overall percentage of 98.7% for the cut-off value of 50%, which indicates that the model classifies those who consider themselves to have a self-perception on the problematic use of the smartphone as well as those who do not equally well.

Thus, the equation is:

Y (self-perception on the problematic use of smartphone of students between 21 and 23 years) = −134.75 + 1.32Tolerance + 1.34Escape + 1.59Disconnection + 0.77Anxiety + 1.95Consequences + 1.94Motivations.(7)

For university students between the ages of 24 and 26, the results of the explanatory self-perception model had the following aspects (see Table 11): of the 546 students, 442 answered No (81%) and 104 Yes (19%). The goodness-of-fit of the best fitting model obtained the following values: a -2log of likelihood = 56.168; a Cox and Snell R^2^ = 0.581; and a Nagelkerke R^2^ = 93.4% (0.934); with the Hosmer and Lemeshow test obtaining a value of 0.876, which demonstrates a good fit, as its significance is ≥0.05 [38]. The specificity and sensitivity were high (99.1% and 94.2%, respectively), with an overall percentage of 98.2% for the cut-off value of 50%, indicating that the model classifies those who consider themselves to have a self-perception on the problematic use of the smartphone as well as those who do not, equally well.

Thus, the equation is:

Y (self-perception on the problematic use of smartphone of students between 24 and 26 years) = −77.27 + 1.06Tolerance + 0.92Escape + 0.79Disconnection + 0.47Anxiety + 1.06Consequences + 0.83Motivations.(8)

Table 12 shows the results of the explanatory model of students older than 26 (age selection variable). Of the 1366 students, 1088 answered No (79.6%) and 278 Yes (20.4%). The goodness-of-fit of the best fitting model obtained the following values: a -2log of likelihood = 86.276; a Cox and Snell R^2^ = 0.612; and a Nagelkerke R^2^ = 96.3% (0.963); with the Hosmer and Lemeshow test obtaining a value of 0.987, which demonstrates a good fit, as its significance is ≥0.05 [38]. Both the specificity and its sensitivity were high (99.2% and 96.4%, respectively), with an overall percentage of 98.6% for the cut-off value of 50%, which indicates that the model classifies those who consider themselves to have a self-perception on the problematic use of the Smartphone as well as those who do not, equally well.

Thus, the equation is:

Y (self-perception on the problematic use of smartphone of students older than 26 years) =−117.61 + 1.72Tolerance + 1.56Escape + 1.49Disconnection + 0.75Anxiety + 1.48Consequences + 1.22Motivations(9)

On Table 12, it is observed that the factors that appeared in the MPPUSA significantly intervened on the explanation; However, having in mind its relevance (B value), it was observed that the order was different from the general model and the order found for different ages, being Tolerance; Escape; Disconnection; Consequences; Motivations; Anxiety for those over 26 years old.

## 4. Discussion

We are in agreement with [39] in that the technologies themselves are not elements that can provoke a problematic behavior in its use, but it is the individuals who develop this behavior, which may or may not be problematic [9].

In this sense, after utilizing the MPPUSA, the results obtained provided us with a profile of a young Spanish and Colombian university student who has a problematic behavior with the mobile device as a function of 6 factors relative to the tolerance towards the time spent using the device, its regard as a means of escape or the disconnection to the world that surrounds them, linked to the anxiety that could be caused if they do not use it, just as the results reached by [17], who showed that the university students with whom the same instrument was utilized, felt better when they utilized the smartphone to evade or avoid situations they found themselves in. The results also showed that the participating students felt that not having the device available gave them anxiety and stress, the negative consequences its use could have due to their using it most of their time in different ways, and lastly, the social motivations related to its use of not, in line with data from [12,17,18,34]. It is meaningful that these results coincide with those from [40], who reached similar results after the use of an instrument called Questionnaire of Experiences Related to the Mobile Phone (Spanish acronym: CERM) with university students, although its short length (10 items) did not analyze in detail aspects such as feelings of avoidance or FOMO.

Taking into account the objectives established, it was verified that the students self-perceived not having a problematic use of the device. This result should be pondered, given that the study by [13] pointed that the university students have control over the addiction to the Internet (85.17%), and in our case, the perception of not having a problematic use was 79.9%. This shows that the university students younger than 35 years old do not consider a problematic or addictive use of the Smartphone or the Internet.

As opposed to results reached by [17,18,21], after the use of the same instrument along the same line, and also as opposed from results reached by [41], who reported an increasing trend of cell phone use after the use of MMPUS. Nevertheless, it is significant that in general, the Colombian students were more aware of the negative consequences that its use could imply, pointing out that this is linked to the time they spend connected or using it in a generalized manner or having to hide this usage time from family and friends, data that coincides with those from [42], who after the application of the HUTL, pointed out the same aspects for this study population. They are also more aware that their state of rest and their academic performance will the affected due to the excessive use of the Smartphone [18,43], with this aspect being common to all the research studies performed with other instruments such as MMPUS, CERM or HUTL [17,40,41,42,43]. Nevertheless, these behaviors that are detrimental, in one way or another, to daily life, are also observed in the use of substances o addiction to the Internet, where the study by [44] points that the altered behaviors related to the Internet, taking into account the sex of the students, could be due to the use or contents consumed. Therefore, we should ask ourselves if the devices are understood as mere devices or associated to the consumption of contents they enjoy.

They also indicated that when they are bored, sad or alone, they use the mobile phone. On other hand, they also commented that if they did not have a device such as this, it would be difficult for their friends to locate them and that they did not like it when the turned their phones off, and this is where the importance a smartphone has in their social relations is derived from just as the results found after the use of the MMPUS [42]. On the other hand, the Spanish students have features of the FOMO syndrome, manifesting that they are worried about not having the device with them, and they would miss calls or messages, and that it is difficult for them to turn the phone off [12,18,29,43].

If we center our attention to the age of the participants, it is verified, as in the work by [12], that the younger students from both countries had a PSU as compared to those who were older, as opposed to the results from [45], given that significant differences were found in the factors found that referred to the use of the mobile phone as a means of escape, to disconnect, anxiety and the negative consequences due to its use.

Gender could be pointed out as an element that determines the attitude towards the mobile phone [32,43] an aspect that coincides with other tests applied [40,41,42]. As opposed to the work by [12,41,42,43], it was the women, and more specifically Spanish women, the ones who had a problematic attitude with the device, such as the shown in the results from works by [18,20,46,47,48,49], as the participating sample expressed feeling anxiety and disconnection with their surroundings if they did not have a working phone [47,48,49,50].

On the other hand, if attention is placed on the macro area of study, as referred to the awareness of the consequences, the Arts and Humanities students, as well as the Engineering and Architecture ones, as compared to the other macro areas, such in the case of Social Sciences, are ones who had features of the so-called FOMO [10,29,45]. As for the elements that comprise the factor Tolerance, it was verified that the students from the macro areas of Experimental Sciences, Engineering and Architecture, were more aware of having less tolerance (being in a bad mood, using the device due to boredom or for feeling alone, and to make superfluous calls) [46,50]. It should be highlighted that the students from the area of Health Sciences did not have any prevalence of problematic behavior.

With respect to the factors reached in this study, it can be confirmed that the previous works [30,34,49,50] have shown the existence of 6 factors, and that the grouping of the items, aside from the correlations between themselves, determine the elements that define a problematic behavior towards smartphones, and about which work models should be developed to be able to perform an intervention.

Lastly, it was verified that the model of 6 factors created in line with the one from [35], follows the trail of the data from [17,30,49,50], and points to the Colombian students being closer to this model as compared to the Spanish students, as it describes the factor Consequences as being more significant in the model, as compared to Escape from the Spanish group. On the other hand, age and sex do not exactly follow the general model.

The section should not end without indicating the limitations of this international study, where the Latin American context, as well as the geography of the terrain, could define uses, trends, and views. One of these limitations could language used. Although in this case the language used was Spanish, it should be point out that just as with other languages, it has local variations depending on the country we find ourselves in. Therefore, the initial instrument developed in the Spanish utilized in Spain had to be adapted to Colombian Spanish, resulting in a delay of the research study.

On the other hand, although the sample was large, the Spanish representation should be increased, not only for the generalization of the data, but also for the performing a comparison that is not catalogued as cross-sectional, and that contributes a PSU analysis model for the entire Latin American context, which entails the intention of widening the study to other countries in the American continent.

## 5. Conclusions

Based on the above, it can be concluded that on the one hand, women in general, and in particular the Spanish women who studied within the macro area of Social Sciences, were the ones who had a problematic use of the mobile phone as compared to the men in both countries, showing the same factors as a pharmacological addiction. On the other hand, the students who resided in Colombia utilized the smartphone as a means of escape from boredom. These same students are aware of the consequences of the excessive use of the device on their personal and academic life. Also, the younger Spanish students had symptomatic signs of the FOMO syndrome. Along this line, in both countries, the students who showed these signs were enrolled in the area of Social Sciences. With respect to the Experimental Sciences and Engineering and Architecture students, these recognized spending too many hours being preoccupied with the phone. It should be pointed out that in the macro areas of Arts and Humanities, and Engineering and Architecture, their students were aware of the negative consequences of its use on their personal and academic lives.

Ultimately, it should be indicated, specifically for the differences found according to age and the country of origin, that the younger students, independently of the country, had a problematic use of smartphones and that Colombian students were closer to the general model, while for the Spanish ones, the order of relevance of each of the factors varied, while the general model does not fit the age or sex of the students.

## Figures and Tables

**Table 1:** Profile of the students as a function of the macro area, age and country.

				Macro Area						
				SJS	HS	AH	ES	EA	TOTAL	TOTAL Country
Country	Colombia	Age	From 18 to 20 y.o.	3.5%	4.5%	1.5%	1.0%	6.5%	16.9%	74.0%
			From 21 to 23 y.o.	3.2%	3.1%	0.8%	0.5%	5.0%	12.6%	
			From 24 to 26 y.o.	3.6%	2.4%	0.4%	0.7%	4.4%	11.5%	
			Older than 26	11.4%	8.1%	1.4%	2.0%	10.1%	33.0%	
	Spain	Age	From 18 to 20 y.o.	13.9%	0.0%	0.0%	0.0%	0.0%	13.9%	26.0%
			From 21 to 23 y.o.	8.9%	0.0%	0.0%	0.0%	0.0%	8.9%	
			From 24 to 26 y.o.	2.1%	0.0%	0.0%	0.0%	0.0%	2.1%	
			Older than 26	1.1%	0.0%	0.0%	0.0%	0.0%	1.1%	
			TOTAL	47.7%	18.1%	4.1%	4.2%	25.9%	100%	

Notes: SJS = Social and Judicial Sciences; HS = Health Sciences; AH = Arts and Humanities; ES = Experimental Sciences; EA = Engineering and Architecture.

**Table 2:** Frequency distribution of the items from the MPPUSA Questionnaire.

MPPUSA Items	*n*	M	SD
I can never spend enough time on my mobile phone	4009	2.61	1.279
I have used my mobile phone to make myself feel better when I was feeling down	4009	2.83	1.423
I find myself occupied on my mobile phone when I should be doing other things, and it causes problems	4009	2.82	1.413
I have tried to hide from others how much time I spend on my mobile phone	4009	1.77	1.209
I lose sleep due to the time I spend on my mobile phone	4009	3.06	1.457
I have spent with the mobile phone more than I should have	4009	2.36	1.421
When out of range for some time, I become worried about the thought of missing a call	4009	2.81	1.442
Sometimes, when I am on my mobile phone and I am doing other things, I get carried away with the conversation and I don’t pay attention to what I am doing	4009	2.78	1.368
The time I spend on my mobile phone has increased over the last 12 months	4009	3.54	1.269
I have used my mobile phone to talk to others when I was feeling isolated	4009	3.76	1.301
I have attempted to spend less time on my mobile phone but am unable to	4009	2.23	1.343
I find it difficult to switch off/switch to silent my mobile phone	4009	2.67	1.518
I feel anxious if I have not checked for messages or switched on my mobile phone for some time	4009	2.37	1.391
I have frequent dreams about my mobile phone	4009	1.39	.973
My friends and family complain about my use of the mobile phone	4009	2.45	1.406
If I don’t have a mobile phone, my friends would find it hard to get in touch with me	4009	3.62	1.287
My academic performance has decreased as a direct result of the time I spend on my mobile phone	4009	2.15	1.333
I have aches and pains that are associated with my mobile phone use	4009	2.08	1.336
I find myself using on my mobile phone for longer periods of time than intended	4009	2.86	1.406
There are times when I would rather use my mobile phone than deal with other more urgent matters	4009	2.11	1.364
I am often late for appointments because I’m talking on my mobile phone when I shouldn’t be	4009	1.80	1.245
I become irritable if I have to switch off/to silent my mobile phone for classes, meals, or at the cinema	4009	1.56	1.086
I have been told that I spend too much time on my mobile phone	4009	2.69	1.445
More than once I have been in trouble because my mobile phone has gone off during a class, at the cinema, or in a theatre	4009	2.37	1.438
My friends don’t like it when my mobile phone is switched off/to silent	4009	2.84	1.410
I feel lost without my mobile phone	4009	2.43	1.420

Notes: M = mean; SD= standard deviation. Source: Author created.

**Table 3:** Results of the bivariate correlations of the items from the 6 dimensions of the questionnaire.

		T	E	D	A	C	M
**T**	r	1.000	0.443 **	0.466 **	0.444 **	0.592 **	0.307 **
	*p*		0.000	0.000	0.000	0.000	0.000
**E**	r		1.000	0.473 **	0.495 **	0.598 **	0.328 **
	*p*			0.000	0.000	0.000	0.000
**D**	r			1.000	0.754 **	0.589 **	0.354 **
	*p*				0.000	0.000	0.000
**A**	r				1.000	0.678 **	0.314 **
	*p*					0.000	0.000
**C**	r					1.000	0.334 **
	*p*						0.000
**M**	r						1.000
	*p*						

Note: T = Tolerance; E = Escape; D = Disconnection; A = Anxiety; C = Consequences; M = Motivations. ** The correlation is significant at 0.01 (2-tailed).

**Table 4:** Binary logistic regression of the self-perception on the problematic smartphone use ^a^ of Spanish and Colombian university students with respect to the MPPUSA.

	B	E.T.	Wald	gl	Sig.	Exp (B)	C.I. 95% for EXP(B)	
							Lower	Upper
Tolerance	1.129	0.122	86.236	1	0.000	3.093	2.437	3.926
Escape	1.105	0.107	106.641	1	0.000	3.020	2.448	3.724
Disconnection	1.115	0.106	110.914	1	0.000	3.051	2.479	3.755
Anxiety	0.540	0.077	49.069	1	0.000	1.715	1.475	1.995
Consequences	1.179	0.091	166.350	1	0.000	3.252	2.718	3.890
Motivations	0.987	0.112	77.352	1	0.000	2.683	2.153	3.343
Constant	−89.073	6.691	177.220	1	0.000	0.000		

Note. ^a^ = Dependent variable: Self-perception of having a problematic use of the smartphone Yes and No.

**Table 5:** Binary logistic regression of the self-perception of the problematic smartphone use ^a^ of Colombian university students with respect to the MPPUSA.

	B	E.T.	Wald	gl	Sig.	Exp (B)	C.I. 95% for EXP(B)	
							Lower	Lower
Tolerance	1.124	0.131	73.716	1	0.000	3.077	2.381	3.977
Escape	1.062	0.113	87.622	1	0.000	2.892	2.315	3.612
Disconnection	1.089	0.114	91.520	1	0.000	2.970	2.376	3.712
Anxiety	0.495	0.080	37.976	1	0.000	1.640	1.401	1.919
Consequences	1.191	0.103	133.351	1	0.000	3.291	2.688	4.028
Motivations	0.952	0.121	61.350	1	0.000	2.590	2.041	3.286
Constant	−88.133	7.340	144.163	1	0.000	0.000		

Note. ^a^ = Dependent variable: Self-perception of having a problematic use of the Smartphone Yes and No.

**Table 6:** Binary logistic regression of the self-perception on the problematic smartphone use ^a^ of Spanish university students with respect to the MPPUSA.

	B	E.T.	Wald	gl	Sig.	Exp (B)	C.I. 95% for EXP(B)	
							Lower	Lower
Tolerance	1.461	0.399	13.394	1	0.000	4.309	1.971	9.422
Escape	1.475	0.354	17.312	1	0.000	4.369	2.181	8.751
Disconnection	1.084	0.306	12.516	1	0.000	2.957	1.622	5.392
Anxiety	1.247	0.347	12.923	1	0.000	3.478	1.763	6.863
Consequences	1.266	0.232	29.758	1	0.000	3.548	2.251	5.593
Motivations	1.461	0.367	15.821	1	0.000	4.312	2.099	8.859
Constant	−108.940	19.958	29.795	1	0.000	0.000		

Note. ^a^ = Dependent variable: Self-perception of having a problematic use of the smartphone Yes and No.

**Table 7:** Binary logistic regression of the self-perception of the problematic Smartphone use ^a^ of male university students with respect to the MPPUSA.

	B	E.T.	Wald	gl	Sig.	Exp (B)	C.I. 95% for EXP(B)	
							Inferior	Superior
Tolerance	1.258	0.210	35.838	1	0.000	3.517	2.330	5.308
Escape	1.223	0.175	48.813	1	0.000	3.397	2.411	4.787
Disconnection	1.164	0.179	42.275	1	0.000	3.204	2.256	4.552
Anxiety	0.598	0.121	24.422	1	0.000	1.819	1.435	2.307
Consequences	1.216	0.153	63.448	1	0.000	3.373	2.501	4.550
Motivations	0.899	0.174	26.843	1	0.000	2.457	1.749	3.453
Constant	−93.506	11.329	68.122	1	0.000	0.000		

Note. ^a^ = Dependent variable: Self-perception of having a problematic use of the smartphone Yes and No.

**Table 8:** Binary logistic regression of the self-perception on the problematic smartphone use ^a^ of female university students with respect to the MPPUSA.

	B	E.T.	Wald	gl	Sig.	Exp (B)	C.I. 95% for EXP(B)	
							Lower	Lower
Tolerance	1.086	0.154	49.572	1	0.000	2.963	2.190	4.010
Escape	1.029	0.140	54.110	1	0.000	2.799	2.127	3.682
Disconnection	1.113	0.138	64.931	1	0.000	3.045	2.323	3.992
Anxiety	0.483	0.103	21.964	1	0.000	1.621	1.324	1.983
Consequences	1.194	0.120	98.472	1	0.000	3.301	2.607	4.179
Motivations	1.081	0.152	50.779	1	0.000	2.947	2.189	3.967
Constant	−88.400	8.595	105.794	1	0.000	0.000		

Note. ^a^ = Dependent variable: Self-perception of having a problematic use of the smartphone Yes and No.

**Table 9:** Binary logistic regression of the self-perception of the problematic smartphones use ^a^ of university students aged between 18 and 20 years old with respect to the MPPUSA.

	B	E.T.	Wald	gl	Sig.	Exp (B)	C.I. 95% for EXP(B)	
							Inferior	Superior
Tolerance	0.875	0.179	23.795	1	0.000	2.399	1.688	3.410
Escape	0.951	0.159	36.032	1	0.000	2.590	1.898	3.533
Disconnection	0.988	0.153	41.600	1	0.000	2.686	1.989	3.627
Anxiety	0.490	0.117	17.473	1	0.000	1.632	1.297	2.054
Consequences	1.034	0.129	64.056	1	0.000	2.813	2.184	3.625
Motivations	0.829	0.168	24.417	1	0.000	2.292	1.649	3.185
Constant	−76.999	9.391	67.220	1	0.000	0.000		

Note. ^a^ = Dependent variable: Self-perception of having a problematic use of the smartphone Yes and No.

**Table 10:** Binary logistic regression of the self-perception on the problematic smartphone use ^a^ of university students between 21 to 23 years of age with respect to the MPPUSA.

	B	E.T.	Wald	gl	Sig.	Exp (B)	C.I. 95% for EXP(B)	
							Lower	Lower
Tolerance	1.323	0.382	12.004	1	0.001	3.753	1.776	7.931
Escape	1.339	0.338	15.669	1	0.000	3.816	1.966	7.406
Disconnection	1.594	0.362	19.439	1	0.000	4.924	2.424	10.003
Anxiety	0.770	0.226	11.617	1	0.001	2.159	1.387	3.361
Consequences	1.952	0.446	19.174	1	0.000	7.044	2.940	16.876
Motivations	1.939	0.560	12.001	1	0.001	6.952	2.321	20.821
Constant	−134.749	29.206	21.286	1	0.000	0.000		

Note. ^a^ = Dependent variable: Self-perception of having a problematic use of the smartphone Yes and No.

**Table 11:** Binary logistic regression of the self-perception on the problematic smartphone use ^a^ of university students between 24 to 26 years of age with respect to the MPPUSA.

	B	E.T.	Wald	gl	Sig.	Exp (B)	C.I. 95% for EXP(B)	
							Lower	Lower
Tolerance	1.058	0.257	16.939	1	0.000	2.882	1.741	4.771
Escape	0.922	0.247	13.943	1	0.000	2.514	1.550	4.078
Disconnection	0.790	0.246	10.282	1	0.001	2.203	1.359	3.569
Anxiety	0.472	0.191	6.071	1	0.014	1.603	1.101	2.332
Consequences	1.060	0.203	27.378	1	0.000	2.885	1.940	4.291
Motivations	0.829	0.295	7.875	1	0.005	2.291	1.284	4.088
Constant	−77.268	14.469	28.517	1	0.000	0.000		

Note. ^a^ = Dependent variable: Self-perception of having a problematic use of the smartphone Yes and No.

**Table 12:** Binary logistic regression of the self-perception on the problematic smartphone use ^a^ of students older than 26 years old with respect to the MPPUSA.

	B	E.T.	Wald	gl	Sig.	Exp (B)	C.I. 95% for EXP(B)	
							Lower	Lower
Tolerance	1.721	0.346	24.689	1	0.000	5.591	2.836	11.024
Escape	1.562	0.285	30.009	1	0.000	4.768	2.727	8.337
Disconnection	1.486	0.275	29.122	1	0.000	4.418	2.576	7.579
Anxiety	0.747	0.193	14.941	1	0.000	2.111	1.445	3.083
Consequences	1.476	0.241	37.435	1	0.000	4.375	2.727	7.019
Motivations	1.222	0.229	28.590	1	0.000	3.394	2.169	5.312
Constant	−117.614	18.738	39.397	1	0.000	0.000		

Note. ^a^ = Dependent variable: Self-perception of having a problematic use of the smartphone Yes and No.
